# Safety and Efficiency of Rotational Atherectomy in Chronic Total Coronary Occlusion—One-Year Clinical Outcomes of an Observational Registry

**DOI:** 10.3390/jcm12103510

**Published:** 2023-05-17

**Authors:** Mohamed Ayoub, Noé Corpataux, Michael Behnes, Tobias Schupp, Jan Forner, Ibrahim Akin, Franz-Josef Neumann, Dirk Westermann, Volker Rudolph, Kambis Mashayekhi

**Affiliations:** 1 Division of Cardiology and Angiology, Heart Center University of Bochum, 32545 Bad Oeynhausen, Germany; 2 Department of Cardiology, Bern University Hospital, University of Bern, 3010 Bern, Switzerland; 3 Department of Cardiology, Angiology, Haemostaseology and Medical Intensive Care, University Medical Centre Mannheim, Medical Faculty Mannheim, 68167 Mannheim, Germany; 4 Department of Cardiology and Angiology II, University Heart Center Freiburg, 79189 Bad Krozingen, Germany; 5 Department of Internal Medicine and Cardiology, Mediclin Heart Centre Lahr, 77933 Lahr, Germany

**Keywords:** coronary chronic total occlusion, CTO, rotational atherectomy, percutaneous coronary intervention, coronary artery disease

## Abstract

The study sought to assess the procedural success of rotational atherectomy (RA) in coronary chronic total occlusion (CTO) and to investigate the in-hospital and one-year outcomes following RA. From 2015 to 2019, patients undergoing percutaneous coronary intervention for CTO (CTO PCI) were retrospectively included into the hospital database. The primary endpoint was procedural success. Secondary endpoints were in-hospital and one-year major adverse cardiovascular and cerebral event (MACCE) rates. During the study period of 5 years, 2.789 patients underwent CTO PCI. Patients treated with RA (*n* = 193, 6.92%) had a significantly higher procedural success (93.26% vs. 85.10%, *p* = 0.0002) compared to those treated without RA (*n* = 2.596, 93.08%). Despite a significantly higher rate of pericardiocentesis (3.11% vs. 0.50%, *p* = 0.0013) in the RA group, the in-hospital and one-year MACCE rate was similar in both groups (4.15% vs. 2.77%, *p* = 0.2612; 18.65% vs. 16.72%, *p* = 0.485). In conclusion, RA is associated with higher procedural success for CTO PCI, but has higher risks for pericardial tamponade than CTO PCI without the need for RA. Nevertheless, in-hospital and one-year MACCE rates did not differ in-between both groups.

## 1. Introduction

The prevalence of coronary chronic total occlusion (CTO) is reported in approximately 15% of all patients undergoing percutaneous coronary interventions (PCI) [1,2]. Due to the aging population and the ever-increasing number of coronary angiograms carried out each year throughout the world, more and more severely calcified lesions are observed and treated. Calcified occlusions are more complex and have been linked with high rates of procedural failure and adverse clinical outcomes [3]. Balloon uncrossable and undilatable CTO lesions, represented in 9–12% of all CTOs, are the most challenging scenarios in calcified CTOs [4,5,6,7,8]. While four different crossing strategies have been described according to wiring direction (antegrade or retrograde) and whether or not an extra plaque wire passage was used (intraplaque wiring versus dissection and re-entry strategies), those challenging lesions often require an atherectomy device for lesion passage and plaque preparation [9,10]. Rotational atherectomy (RA) has been reported to be the most suitable device for this lesion subset [11,12,13,14]. Despite the high prevalence of CTOs, data on RA during CTO PCI are mostly limited to registries with small sample sizes and missing follow-up data [7,15,16,17,18]. The percentage of CTO treated with RA varies substantially in the different studies, mainly due to the varying expertise of the different centers and operators.

Therefore, the present study aims to investigate the periprocedural success and one-year outcomes for CTO PCI with and without RA using a large-scaled database from a high-volume CTO center.

## 2. Materials and Methods

### 2.1. Study Population

All consecutive patients undergoing CTO PCI between January 2015 and December 2019 were retrospectively entered into the hospital database. There were no formal exclusion criteria and all patients who provided informed consent were included consecutively. In this single-centre analysis, the patients were stratified according to whether RA was performed or not during CTO PCI. RA was performed with the Rotablator system (Boston Scientific Corp., Natick, MA, USA) [19]. The majority of patients with chronic coronary syndrome received clopidogrel and aspirin after drug eluting stent (DES) implantation. The recommended DAPT duration was 12 months for all acute or chronic coronary syndrome patients until August 2017, excepting the presence of concomitant oral anticoagulation or high-bleeding-risk features. From August 2017, the routinely recommended DAPT duration for chronic coronary syndrome patients became 6 months, in agreement with existing European guidelines [20].

### 2.2. Definitions

CTOs were defined based on the CTO-ARC criteria (a total occlusion, documented to be ≥3-month-old, with absence of antegrade flow through it and no thrombus, no staining at the proximal cap, and presence of mature collaterals) [9]. Calcification grade was defined by angiography as mild (spots), moderate (involving ≤50% of the reference lesion diameter), or severe (involving >50% of the reference lesion diameter). Technical success was defined as residual stenosis <30% with antegrade TIMI 3 flow in the CTO target vessel [10]. Procedural success was defined as technical success in the absence of in-hospital major adverse cardiovascular and cerebral events (MACCE). In-hospital and one-year MACCE included any of the following adverse events before hospital discharge and at one-year follow-up, respectively: all-cause death, type 4 myocardial infarction (MI) using the Third Universal Definition of Myocardial Infarction [21], stroke, target-vessel revascularization (TVR) or target-lesion revascularization (TLR) with PCI or coronary artery bypass graft (CABG), and tamponade requiring pericardiocentesis.

### 2.3. Follow-Up

As part of the quality management program of our institution, baseline demographic, clinical, angiographic, and procedural data, as well as outcome data, were entered routinely into the hospital monitoring database. As part of our routine follow-up, we performed an interview 1 year after any PCI and the results were documented as well in the database. Written informed consent for PCI was obtained from each patient and the collection of data was performed in accordance with the Declaration of Helsinki and approved by the institutional review board (Ethical approval number: EK 21-1100).

### 2.4. Clinical Endpoints

The primary endpoint was the procedural success in CTO PCI. The secondary endpoints were the in-hospital and the one-year MACCE.

### 2.5. Statistical Methods

Baseline and procedural characteristics, as well as lesion characteristics, are shown with means and standard deviations or medians (interquartile range, IQR) unless otherwise specified and were compared between the CTO with RA group and the CTO without RA group using the student t-test. Categorical variables were expressed as percentages and were compared using Pearson’s chi-square test or Fisher’s exact test. Multivariable analyses were calculated at baseline for the risk of all-cause MACCE by using Cox regression with backward elimination. Crude and adjusted hazard ratios with 95% confidence intervals (95% CI) were calculated after the selection of the confounding variables based on the grounds of univariable association with the given endpoints in the present study (*p* < 0.05). Cumulative event rates were calculated according to the Kaplan–Meier method, and comparisons were performed with the log-rank test. A *p* value of <0.05 was considered statistically significant, and all *p* values were 2-sided. All statistical analyses were performed with JMP 13.0 (SAS, Cary, NC, USA).

## 3. Results

A total of 2.789 patients treated with CTO PCI were enrolled during the study period, of whom 193 (6.92%) underwent additional RA (CTO with RA group). The principal baseline characteristics of the CTO with RA group and the non-RA group (CTO without RA group) are listed in Table 1. The population in the atherectomy group was older on average (70.33 ± 8.97 vs. 65.73 ± 10.84 years, *p* < 0.0001) and had more comorbidities such as diabetes (43.41% vs. 29.85%, *p* = 0.0002) and hypertension (92.51% vs. 85.86%, *p* = 0.0081). The number of patients with prior CABG was higher in the RA group (33.52% vs. 16.06%, *p* = 0.0001), while the number of patients with prior MI was similar in both groups (38.35% vs. 34.50%, *p* = 0.3290).

The specific lesion characteristics are shown in Table 2 and the procedural characteristics are outlined in Table 3. The lesions in the RA group were more calcified (*p* < 0.0001) and had higher intra-lesion angulation (*p* < 0.0001). The CTO lesions treated with RA needed more stents (2.19 ± 1.15 vs. 1.73 ± 1.20, *p* <0.0001) with an overall larger diameter (4.16 ± 0.53 vs. 3.17 ± 0.04 mm, *p* 0.05) than the lesion treated with conventional CTO PCI technique. Approximately 30% of the CTO were performed using the retrograde approach, irrespective of the use of RA. We applied more inflation pressure in the RA group both in the pre-dilatation and in the post-dilatation phase (*p* < 0.0001). The PCI procedure in the RA group took longer fluoroscopic times (*p* < 0.001) and used more contrast medium volumes (*p* = 0.0002), as well as higher radiation doses (*p* < 0.001).

The primary endpoint (i.e., procedural success) occurred significantly more frequent in the CTO with RA group (93.26% vs. 85.10%, *p* = 0.0002). Accordingly, technical success was achieved more prevalently in the RA group (97.41% vs. 87.87%, *p* < 0.0001) (Table 4).

**Figure 1:**
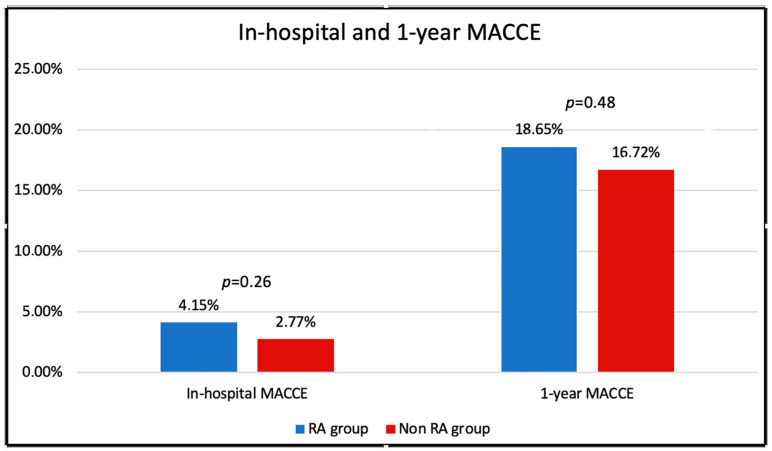
In-hospital and one-year MACCE classified according to rotational atherectomy use.

There were no significant differences among the two study groups with regard to the secondary clinical endpoints. Despite significantly higher rates of tamponades leading to pericardiocentesis (3.11% vs. 0.50%, *p* = 0.0013), the in-hospital MACCE rate was similar in both groups (4.15% vs. 2.77%, *p* = 0.2612). At one year, a total of 491 MACCE events occurred, including 434 (16.72%) and 36 (18.65%) in the RA and non-RA group, respectively (*p* = 0.48) (Figure 1).

**Figure 2:**
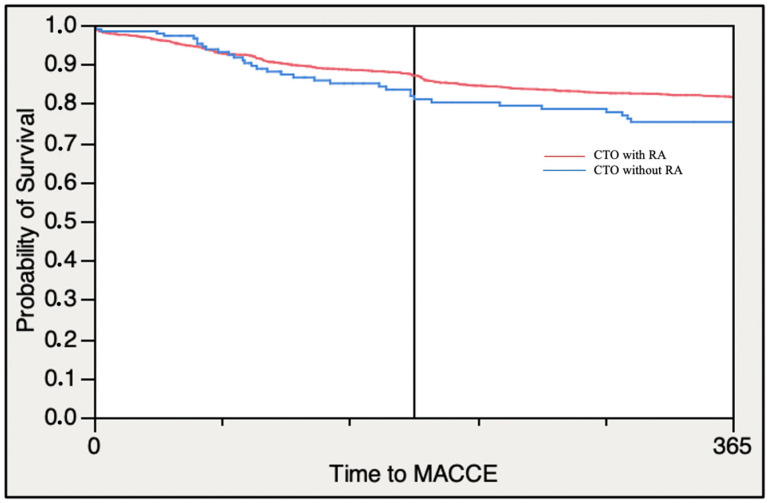
Kaplan–Meier analysis for one-year MACCE according to rotational atherectomy use.

Consistent results were observed regarding the one-year mortality of any causes (3.70% vs. 5.70%, *p* = 0.1625) and one-year stroke rate (0.65% vs. 0.00%, *p* = 0.6256). The Kaplan-Maier curve for one-year MACCE is shown in the Figure 2.

**Figure 3:**
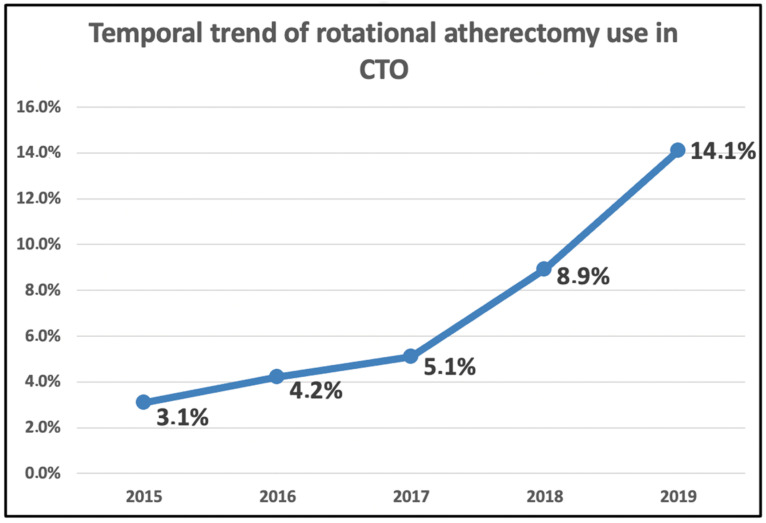
Temporal evolution of rotational atherectomy use for chronic total occlusion intervention during the study period.

In our center, the RA use for CTO increased consistently from 2015 to 2019 (Figure 3).

## 4. Discussion

**Figure 4:**
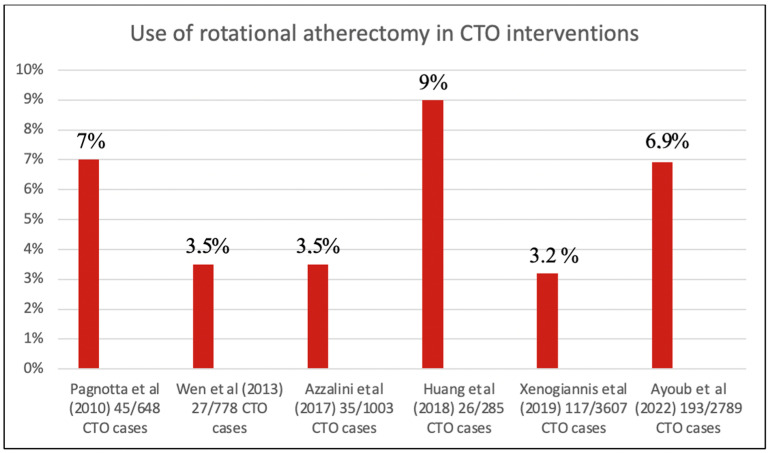
Percentage of CTO lesions treated with rotational atherectomy in previous studies [7,15,16,17,18].

As shown in Figure 4, the presents study represents the largest ever published cohort of CTO patients treated with RA including a one-year clinical follow-up. Multiple trials demonstrated the prognostic and symptomatic benefits of recanalization of CTO lesions improving survival rate, angina symptoms and heart function [22,23,24]. Furthermore, a successfully revascularized CTO was shown to improve long-term survival as compared to failed revascularization within previous studies [25,26].

Our key finding can be summarized as follows:

1. RA is increasingly used in approximately 7% of our CTO cases in the last years, mainly for complex calcified lesions and in patients with prior CABG;
2. RA is linked with a significantly higher rate of procedural and technical success;
3. Patients treated with RA showed similar in-hospital and one-year MACE rates despite a higher rate of tamponade and pericardiocentesis.

In contrast to previous studies, our results are the first to show significantly higher rates of procedural and technical success in CTO patients treated with RA vs. conventional CTO-techniques. In 2019, Xenogiannis and colleagues reported a large atherectomy cohort with 3.607 CTO cases, including 117 (3.2%) in which atherectomy was used with a similar technical (91% vs. 87%, *p* = 0.240) and procedural (90% vs. 85%, *p* = 0.159) success [15]. This is consistent with other trials, with the exception of the study by Azzalini et al., which showed lower procedural success within the 35 patients (3.5%) treated with RA (77% vs. 89%, *p* = 0.04) [18]. While the percentage of lesions treated with RA varies widely in the literature (Figure 4), our data show a steady increase in the use of RA in the last few years (Figure 1). This is partially due to the great expertise and positive experience for plaque modification gained over the past years. There is a cost to using RA, but coronary atherectomy has become an indispensable tool for complex CTO and non-CTO coronary intervention.

Several alternatives to RA (orbital atherectomy, laser atherectomy, or shockwave therapy) have been introduced to clinical practice to enable adequate lesion preparation by modifying the calcified plaque before stent placement. Some of these devices have been used in terms of CTO lesions. Excimer laser coronary atherectomy therapy has been shown as an effective method for the treatment of in-stent restenosis in CTO [27]. Other devices, such as shockwave therapy, can be used in CTO PCI for the treatment of undilatable lesions or further plaque modification, but because of their profile, they do not yet have a role in the treatment of uncrossable lesions.

The technical aspect of CTO PCI is addressed in our study, and we conclude that technical success is higher in the rotating atherectomy group. However, it is important to note that technical and procedural success does not necessarily mean “patient success”. We do see an overall higher perforation rate in the RA group; however, this does not translate into a higher in-hospital or one-year MACCE rate.

Our results should be interpreted in view of several limitations. First, they arise from an observational single center study and, as such, may suffer from limited generalizability. Second, the RA group had higher success rate, despite higher angiographic and clinical complexity. This is probably based on the fact that the Rota Wire has already passed the CTO lesion in most of the cases. Third, the choice of whether to perform a coronary atherectomy was left to the discretion of the operators. Fourth, the Japan-chronic total occlusion (J-CTO) and the Prospective Global Registry of Chronic Total Occlusion Interventions (PROGRESS-CTO) scores are not part of our prospective evaluation focusing on clinical outcome analysis. Furthermore, no information on freedom from angina was available for the present study. Finally, the interventions were performed in a high-volume CTO center by special trained operators, which may restrict extrapolation to less experienced operators and lower volume clinics.

Despite the limitations cited, our study represents the largest register of CTO treated by RA ever published to date. For the first time, CTO treated with RA is shown to be superior to conventional CTO PCI techniques in terms of procedural success. This benefit does not come at the expense of a higher rate of in-hospital or one-year MACCE.

## 5. Conclusions

In patients presenting with CTO and undergoing PCI for revascularisation, we found higher rates of procedural success and no difference in the one-year MACCE rate in patients treated with RA despite a higher risk of tamponade and pericardiocentesis. RA is a very effective and useful tool in the armamentarium of a CTO PCI operator, treating complex calcified CTO lesions.

## Figures and Tables

**Table 1:** Baseline characteristics in patients undergoing CTO PCI with and without RA.

Patient Characteristics	CTO without RA (*n* = 2596)	CTO with RA (*n* = 193)	*p* Value
Age (years)	65.73 ± 10.84	70.33 ± 8.97	<0.0001 *
Men	82.55%	80.83%	0.56
BMI (kg/m^2^)	28.44 ± 4.66	28.19 ± 4.47	0.45
eGFR, mL/min/1.73 m^2^	72.43	66.18	<0.0001 *
CAD presentation			0.37
ACS	12.79%	10.36%	
No ACS	87.21%	89.64%	
Diabetes mellitus	29.85%	43.41%	0.0002 *
Dyslipidemia	89.89%	92.27%	0.37
Hypertension	85.86%	92.51%	0.0081 *
Smoking (current)	20.01%	10.99%	0.0024 *
LVEF (%)			0.29
>51%	62.24%	58.89%	
41–51%	20.31%	25.56%	
30–40%	10.89%	11.11%	
0–29%	6.56%	4.44%	
Family History of CAD	43.58%	37.18%	0.13
Prior Myocardial Infarction	38.35%	34.50%	0.33
Prior CABG	16.06%	33.52%	<0.0001 *
Prior CVD	11.6%	12.3%	0.87
LDL max.	104.75 ± 41.39	91.78 ± 35.71	<0.0001 *

Values are given as percentages of patients and numbers or as mean and standard deviation. ACS = acute coronary syndrome; BMI = body mass index; CABG = coronary artery bypass graft surgery; CAD = coronary artery disease; CTO = chronic total occlusion; CVD = cerebrovascular disease; eGFR = estimated glomerular filtration rate in mL/min/1.73 m^2^; LDL = low-density lipoprotein in mg/dl; LVEF = left ventricular ejection fraction; RA = rotational atherectomy, * statistically significant with *p* < 0.05.

**Table 2:** Lesion characteristics in patients undergoing CTO PCI with and without RA.

	CTO without RA (*n* = 2596)	CTO with RA (*n* = 193)	*p* Value
Target vessel			<0.0001 *
Right coronary	50.35%	55.96%	
Left circumflex	21.34%	19.17%	
Left anterior descending	26.93%	20.73%	
SVG	0.7%	0.0%	
Number of treated vessels	1.35 ± 0.58	1.41 ± 0.59	0.14
Number of treated segments	6.63 ± 5.41	5.67 ± 4.94	0.009 *
Lesion length (mm)			0.15
<10 mm	2.93%	0.52%	0.22
10–20 mm	18.09%	17.28%	
>20 mm	78.98%	82.20%	
Ostial lesion	6.69%	7.2%	0.32
Calcification			<0.0001 *
None	6.49%	0.52%	
Mild	28.42%	2.07%	
Moderate	28.80%	11.40%	
Severe	36.29%	86.01%	
Eccentric calcification	53.12%	49.41%	0.35
Tortuosity	20.74%	23.83%	0.31
Relevant side brunch	29.58%	23.40%	0.072 *
Intra-lesion Angulation			0.13
none	11.98%	11.92%	
<45%	36.47%	30.05%	
45–90%	45.12%	48.19%	
>90%	6.43%	9.84%	<0.0001 *

Values are given as percentages of patients and numbers or as mean and standard deviation. CTO = chronic total occlusion; PCI = percutaneous coronary intervention; RA = rotational atherectomy; SVG = saphenous vein graft, * statistically significant with *p* < 0.05.

**Table 3:** Procedural characteristics in patients undergoing CTO PCI with and without RA.

	CTO without RA (*n* = 2596)	CTO with RA (*n* = 193)	*p* Value
Balloon diameter pre-dilatation, mm	2.50 ± 1.23	2.99 ± 2.74	<0.0001 *
Maximum inflation pressure pre-dilatation, atm	17.63 ± 5.37	22.65 ± 18.59	<0.0001 *
Number of stents implanted	1.73 ± 1.20	2.19 ± 1.15	<0.0001 *
Diameter of implanted stent, max., mm	3.17 ± 0.04	4.16 ± 0.53	0.05 *
Overall stent length, mm	54.18 ± 32.5	50.55 ± 30.21	0.23
Balloon diameter post-dilatation, mm	3.64 ± 2.13	3.96 ± 3.08	<0.0001 *
Post-dilatation pressure, atm	20.12 ± 7.04	21.98 ± 6.92	<0.0001 *
Burr Size used			
1.25 mm		33.14%	
1.50 mm		47.34%	
1.75 mm		16.57%	
2.0 mm		2.96%	
TIMI flow post PCI			0.0063 *
TIMI 0/TIMI I	12.2%	5.79%	
TIMI II	1.48%	1.05%	
TIMI III	86.33%	93.16%	
Access site			0.0027 *
Single Radial access	31.08%	20.32%	
Any Femoral access	68.60%	78.61%	
Guiding catheter size, Fr			<0.0001 *
6	62.82%	32.13%	
7	34.25%	60.62%	
8	2.93%	7.25%	
CTO technique			0.29
antegrade	73.42%	69.95%	
retrograde	26.58%	30.05%	
Procedural time (minutes)	81 (33–126)	127 (94–186)	<0.0001 *
Fluoroscopy time (minutes)	35 (20–60)	54 (35–80)	<0.0001 *
Fluoroscopic Dose Area Product (cGy*cm^2^)	9710 (5388–16,398)	12,881 (4347–20,632)	<0.0001 *
Contrast volume used (mL)	260 (190–390)	310 (200–400)	0.0002 *

Values are given as percentages of patients and numbers or as mean and standard deviation. CTO = chronic total occlusion; PCI = percutaneous coronary intervention; RA = rotational atherectomy; TIMI = thrombolysis in myocardial infarction, * statistically significant with *p* < 0.05.

**Table 4:** Primary and Secondary endpoints % (*n*).

	CTO without RA (*n* = 2596)	CTO with RA (*n* = 193)	*p* Value
one-year MACCE	16.72% (434)	18.65% (36)	0.48
one-year Mortality	3.70% (96)	5.7% (11)	0.16
one-year MI	1.2% (31)	1.04% (2)	0.84
one-year TVR	16.26% (422)	18.65% (36)	0.39
one-year Stroke	0.65% (17)	0,00% (0)	0.63
one-year TLR	14.33% (372)	18.13% (35)	0.16
In-hospital MACCE	2.77% (72)	4.15% (8)	0.26
Mortality	1.04% (27)	1.04% (2)	1.0
MI Type 4a	5.5% (142)	12.5% (24)	<0.0004 *
TVR	16.35% (475)	18.52% (40)	0.40
Stroke	0.19% (5)	0% (0)	1.0
Technical success	87.87% (2281)	97.41% (188)	<0.0001 *
Procedural success	85.10% (2209)	93.26% (180)	0.0002 *
Procedural time (min)	81 (33.6, 126)	127 (94, 186)	<0.0001 *
Fluoroscopy time (min)	35 (20, 60)	54 (35, 80)	<0.0001 *
Contrast volume used (mL)	260 (190, 390)	310 (200, 400)	<0.0002 *
Fluoroscopic Dose Area Product (cGy*cm^2^)	9710 (5388–16,398)	12,881 (4347–20,632)	<0.0001 *
Major Complication % (*n*)			
Perforation	1.99% (51)	7.94% (15)	<0.0001 *
Pericardiocentesis	0.5% (13)	3.11% (6)	0.0013 *

Values are given as percentages of patients and numbers or as mean and standard deviation. CTO = chronic total occlusion; RA = rotational atherectomy; * statistically significant with *p* < 0.05.

## Data Availability

The datasets used and/or analyzed during the current study are available from the corresponding author on reasonable request.
